# A durable response to programmed cell death 1 blockade in a multidrug-resistant recurrent ovarian cancer patient with HLA-B44 supertype: A case report

**DOI:** 10.3389/fimmu.2022.951422

**Published:** 2022-10-06

**Authors:** Xukai Luo, Yating Sun, Jiajia Li, Qidi Jiang, Lei Yuan, Ting Li, Mo Chen, Liangqing Yao

**Affiliations:** ^1^ Department of Gynecology Oncology, Obstetrics and Gynecology Hospital, Fudan University, Shanghai, China; ^2^ Department of Bioinformatics, Precision Scientific (Beijing) CO., Ltd., Beijing, China

**Keywords:** ovarian clear cell carcinoma, platinum resistance recurrence, chemotherapy resistant, immunotherapy, HLA-B44 supertype, PD-L1

## Abstract

Relapsed/refractory ovarian cancer, especially platinum resistance recurrence, remains a major therapeutic challenge. Here, we present the case of a patient with recurrent ovarian clear cell carcinoma (OCCC) who failed to respond to multiline chemotherapy and target therapy but achieved an immune complete response (iCR) with programmed cell death 1 (PD-1) inhibitor treatment. The overall survival (OS) was 59 months, and the recurrence-free survival (RFS) was 34 months after immunotherapy, which was counting. Meantime, molecular testing results revealed that traditional biomarkers for immunotherapy, including PD-L1 expression, microsatellite instability (MSI), and tumor mutational burden (TMB), were negative. HLA-B44 (B*18:01) supertype was confirmed by sequence-based HLA typing. This case raises the possibility that ovarian cancer patients with multidrug resistance may still benefit from PD-1 inhibitor therapy, even if PD-L1 pathology is negative.

## Introduction

Epithelial ovarian cancer (EOC) is one of the most lethal malignancies in the female reproductive system (1). The various subtypes of EOC exhibit histological and genomic heterogeneity. Ovarian clear cell carcinoma (OCCC), accounting for 5–25% of EOC, is an aggressive malignancy with a poor prognosis and often demonstrates resistance to chemotherapy (2). The median survival of platinum-resistant ovarian cancer is 12 months (3). Multidrug resistance is incurable and a major challenge to cancer therapy.

Immune checkpoint inhibitor (ICI) therapies have made striking progress and revolutionized the treatment of cancer, such as melanoma and renal cell carcinomas (4, 5). However, ovarian cancer has demonstrated limited activity (objective response rate [ORR] of ~8–9%) (6, 7) and, currently, has no FDA-approved indication. Although the effect of ICI therapy in OCCC seemed better than in other EOC types, the ORR is still less than 20% (8). Meantime, programmed death-ligand 1 (PD-L1) expression as a predictor of immunotherapy response is imperfect and improved biomarkers of response are needed (9).

As an independent factor in tumor antigen presentation, human leukocyte antigen class I (HLA-I) plays a central role in antigen recognition and adaptive immune responses (10). Most recently, the HLA-I genotype has been linked with immunotherapy efficacy in melanoma (11). However, the functions of the HLA-I genotype in ovarian cancer are rarely reported.

In this study, we present a case study on an OCCC patient who had relapsed after multiline chemotherapy but achieved immune complete response (iCR) with programmed cell death 1 (PD-1) inhibitor treatment. At the time of submission of this manuscript, the patient has survived for more than 34 months after immunotherapy. Her traditional biomarkers for immunotherapy, including PD-L1 expression, microsatellite instability (MSI), and tumor mutational burden (TMB), were negative. This case raises the possibility that the HLA-B44 supertype may be the potential predictor for immunotherapy in OCCC.

## Case presentation

**Figure 1:**
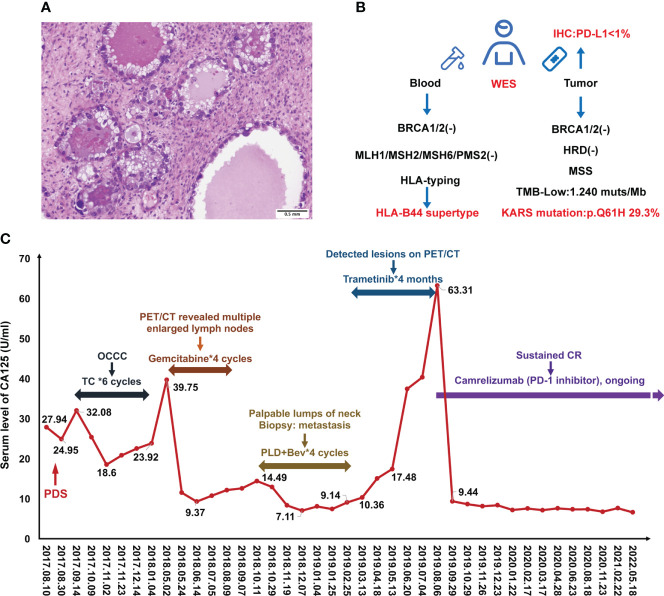
Case presentation during treatment. **(A)** H&E staining of ovarian clear cell carcinoma. **(B)** Scheme of molecular testing strategy. **(C)** Change of serum CA125 levels during the treatment.

A 49-year-old Chinese female was admitted to our hospital due to a sudden persistent abdominal pain in August 2017. The ultrasound revealed a left adnexal complex mass with a moderate amount of ascites and carbohydrate antigen 125 (CA125) value was 29 U/ml (normal level < 35). She underwent comprehensive staging surgery without residual tumor (R0), including hysterectomy, bilateral salpingo-oophorectomy, and systematic lymphadenectomy. The postoperative pathology was diagnosed with OCCC at stage IC2 (T1c_2_N_0_M_0_) **(** **Figure 1A** **)**. Six cycles of adjuvant platinum-based chemotherapy were given according to the TC (paclitaxel [175 mg/m^2^] and carboplatin [AUC = 5], q21d) schedule. She achieved complete response (CR) and kept on regular follow-up with serum CA125 and pelvic-abdominal magnetic resonance imaging (MRI) every 3 months.

Standard subsequent follow-ups were all negative until May 2018, when there was a rise in CA125 to 39.75 U/ml (normal level < 35). PET/CT revealed tumor metastasis at the pelvic and multiple lymph nodes involving the abdominal and thoracic cavities. Considering the disease recurrence just 4 months after the last platinum-containing regimen (platinum-resistant relapse), the patient received four cycles of gemcitabine (1000mg/m^2^, on days 1, 8, 15, q28d). However, her disease did not get controlled. In October 2018, the patient found palpable lumps around her neck of approximately 2 cm in diameter. A biopsy proved metastasis to neck lymph nodes. Given the disease progression, four cycles of pegylated liposomal doxorubicin (PLD) (40 mg/m^2^, q28d) and bevacizumab (10 mg/kg, q28d) were received from October 2018 to Feberuary 2019. During this period, the CA125 levels were below normal **(** **Figure 1C** **)**.

To investigate more effective treatment, tumor tissue and blood samples were tested for the feasibility of target therapies **(** **Figure 1B** **)**. Whole exon sequencing (WES) revealed negative for BRCA1/2 mutations, a microsatellite-stable (MSS) status, KRAS (p.Q61H) mutation, and HLA-B44 (B*18:01) supertype. The tumor mutation burden (TMB) was 1.24 mut/Mb (**Figure 1B**). Less than 1% of the tumor cells was expressed PD-L1 by Immunohistochemistry (IHC). Considering the patient with KRASp.Q61H mutation, trametinib (a MEK inhibitor) (2 mg, qd) was used for 4 months.

**Figure 2:**
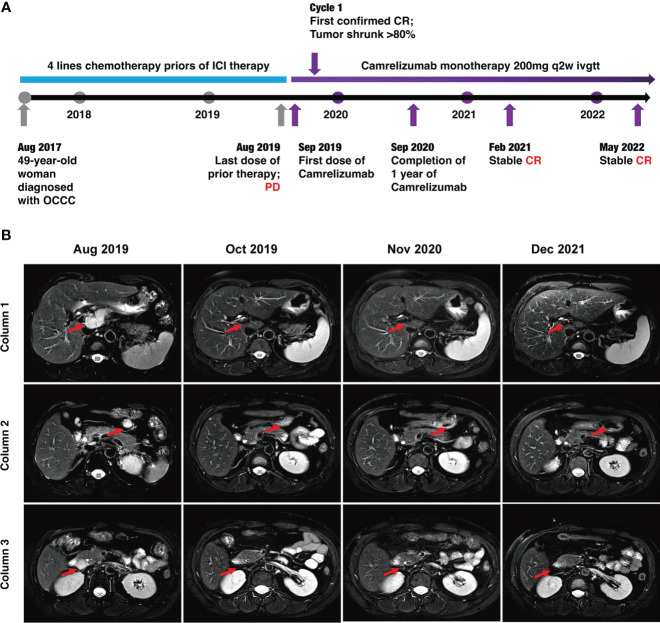
Timeline of the case and MRI findings. **(A)** Timeline of patient diagnosis, prior therapies, and immunotherapy. **(B)** MRI scans demonstrating activity (i.e., complete response) to Camrelizumab. The lymph node metastases were located in front of the inferior vena cava (red arrows) in column 1, anterior to the tail of the pancreas (red arrows) in column 3, and around the head of the pancreas (red arrows) in column 3.

However, during this treatment period, CA125 gradually increased to 63.31 U/ml. Meantime, MRI revealed lymph node metastases located in front of the inferior vena cava (the biggest mass was 4.5*2.0 cm) and other enlarged peripancreatic nodes, which suggested progressive disease (PD) (**Figure 2B**).

Luckily, considering that the patient had HLA-B44 supertype, which has been reported in melanoma, that immunotherapy has the potential to be effective (11). After discussion, the patient and her family agreed to treatment with Camrelizumab (an anti–PD-1 inhibitor). The patient was administered Camrelizumab (200 mg) every 2 weeks (**Figure 2A**). Strikingly, the patient serum CA-125 level dramatically decreased from 63.31 to 9.44 U/ml after one cycle of treatment. The results showed reduced lymph node metastases (short axis < 10 mm) and normal tumor markers, which were suggested as iCR by iRECIST criteria (**Figure 2A**). Considering the inspiring response, anti–PD-1 therapy was conducted from September 2019 until now and the efficacy was assessed as sustained iCR. The radiologic results were recorded over time (**Supplemental Table 1**).

At time of manuscript submission, the patient has had an overall survival (OS) of over 59 months and had no discomfort. The recurrence-free survival (RFS) was 34 months after immunotherapy, and this is a continuing response. The timeline of patient treatment and change of tumor markers is shown in **Figure 2A**. Moreover, the toxicity associated with Camrelizumab treatment was tolerable throughout the whole course of immunotherapy.

## Discussion

This case study provides a description of a PD-L1 negative, MSS, and TMB-L recurrent and multidrug-resistant OCCC patient who was highly sensitive to immunotherapy and had a long and impressive response. Before immunotherapy, the patient suffered the failures of multi-line chemotherapies frequently. When we faced a dilemma in treatment, HLA-B44 (B*18:01) supertype was identified in WES data. In light of this, immunotherapy was considered. After the first cycle of single Camrelizumab administration, the CA125 marker dropped significantly (an approximately 85.09% decrease). The patient luckily benefited from immunotherapy. The PFS was 34 months and the OS was 59 months; this is a continuing response. Importantly, Camrelizumab is an anti–PD-1 monoclonal antibody and monotherapy for this patient, which confirmed the effectiveness of immunotherapy. It is worthwhile to figure out the reasons for the success of the treatment and to reveal potential predictors.

As we know, more than 80% of patients with advanced ovarian cancer will experience a recurrence within 2 years and eventually develop resistance to multiple lines of chemotherapy, which is considered incurable (12). Ovarian cancer patients treated with ICIs did not benefit from the significant response rates seen in other cancers (6, 7). The first study of anti-PD1 nivolumab in platinum-resistant ovarian cancer (*n* = 20) demonstrated an ORR of 15% (13). In the EOC cohort of phase Ib JAVELIN solid tumor study, the response rate to anti–PD-L1 avelumab was similar, estimated at 13.6% in the platinum-resistant sub-group (*n* = 22) (7). In the JAVELIN 200 study, 188 platinum-resistant patients received avelumab alone, with an estimated ORR of 3.7% (14). In a recent phase II clinical trial of pembrolizumab in recurrent ovarian cancer (KEYNOTE-100), the anti–PD-1 antibody pembrolizumab in recurrent ovarian cancer (369 patients) had a low overall response rate across all cohorts (~8%), but the response rate for OCCC (15 patients) was 15.8% (8). There was an agreement that ICIs appear to be a powerful new therapeutic agent for patients with OCCC, which was confirmed by our case. Most of the time, multidrug-resistant recurrence would decrease the confidence of the patients and families, including physicians. Therefore, our case suggested that OCCC patients, even with multiline chemotherapies resistance, may still derive the long-term benefit of immunotherapy.

Several studies have identified several positive predictive markers for ICI therapy, such as PD-L1 overexpression, TMB, and MSI-high (15). However, the positive rates of these biomarkers are very low in ovarian cancer, such as PD-L1 positivity expressions of 30–80% (16, 17), TMB-high of 1.47% (18), and MSI-H of 1.37% (19). Consistently, our case was PD-L1 negative, MSS, and TMB-Low, but the response to immunotherapy showed impressive survival benefits. Therefore, our study suggested that even if these frequently mentioned biomarkers were negative, the patients may still benefit from ICI therapy.

**Figure 3:**
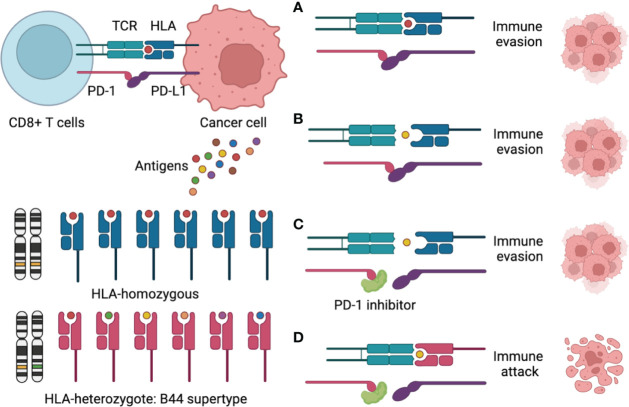
Schematic figure to explain the mechanism of the HLA-B44 supertype influences cancer response to PD-1 inhibitors immunotherapy. **(A)** CD8+ T cells recognize a tumor-associated antigen through the HLA-I/T cell receptor (TCR) complex, but PD-L1 binding to PD-1, which promotes immune evasion; **(B)** CD8+ T cells could not recognize tumor-associated antigens through the HLA-I/TCR complex and PD-L1 binding to PD-1, which promotes immune evasion; **(C)** PD-1 inhibitors overcome immune evasions, but CD8+ T cells could not recognize tumor-associated antigens through the HLA-I/TCR complex, which promotes immune evasion; **(D)** CD8+ T cells recognize many tumor-associated antigens through the HLA—B44/TCR complex, meantime PD-1 inhibitors Inhibit PD-1/PD-L1 interaction, which promotes immune attack.

According to previous research, the impact of HLA-B44 on ICIs survival seemed to be disease specific. B44 was associated with better OS in melanoma (11), but it could be a risk factor in non-small cell lung cancer (NSCLC) (20). Mutation signature can explain the discrepancy. Motif neoepitopes with radical glutamic acid substitutions in the anchor position were associated with improved ICI therapy survival, which was more common in melanoma than in NSCLC (21). However, the role of the HLA type in ovarian cancer immunotherapy is unclear. To our knowledge, only one study in ovarian cancer reported that HLA-B44 was associated with a worse prognosis and more frequent spontaneous antibody responses (22), but its relationship with immunotherapy had never been reported. In this case report, the patient luckily benefited from immunotherapy, and HLA-B44 (B*18:01) supertype was identified using WES. Therefore, we sought to draw a schematic to explain the mechanism (**Figure 3**). In most immunotherapies, CD8^+^ T cells are the main players in killing cancer cells. There are two requirements for CD8^+^ T cell killing tumor cells: First, CD8^+^ T cells recognize tumor-associated antigens through the HLA-I/T cell receptor (TCR) complex; Second, blocking of the PD-1/PD-L1 interaction enhances the direct CD8^+^ T cell killing of tumor cells (23). Therefore, the mechanism for the ICIs response is to activate the immune system, which depends on the antigenicity of the tumor and the efficiency of antigen presentation. Theoretically, greater HLA diversity leads to a greater variety of tumor neoantigens being presented, which could increase the efficacy of treatment with ICIs (24). HLA-B44 is a supertype of HLA-I, which could cross-present new antigens presented by other subtypes of HLA, thus increasing the diversity of HLAs and activating T cells to kill tumor cells (24, 25). Recently, over 50 clinical trials for ovarian cancer were related to immunotherapy. OCCC-focused clinical trials with immunotherapies included durvalumab and nivolumab (NCT03602586, 05026606). However, no HLA genotype-based immunotherapy in ovarian cancer has been reported so far. Further research is needed to figure out the mutation landscape of HLA-B44 and its ability as a predictive marker for response to immunotherapy in ovarian cancer.

Mutational analysis by whole exome sequencing revealed the mutation profile of OCCC, such as ARID1A (40–60%), PIK3C (33–51%), ARID1B (10%), PIK3R1 (7–8%), KRAS (9–17%), TP53 (5–15%), and CTNNB1 (5–10%) (26). KRAS (p.Q61H) mutation was identified in our patient’s samples, which agrees with previous reports. In recent early clinical trials, MET inhibitors had shown preliminary antitumor activity in KRAS-mutation NSCLC (27) and low-grade serous ovarian cancer (28). However, we did not observe this effect in this case. The trametinib, a MET inhibitor, failed to prevent disease progression.

Lynch syndrome-associated ovarian cancer includes OCCC, which was considered a good candidate for treatment with checkpoint inhibitors (29). The patient’s father was diagnosed with rectal cancer. Therefore, Lynch syndrome should be considered. In a previous study, 43% of OCCC tumors expressed PD-L1 and 67% had mismatch repair deficiency, which was the reason why ICI therapy was more sensitive in OCCC (30). However, our patient was PD-L1 negative and MSS. Meanwhile, we re-analyzed the data; neither germline pathogenic/likely pathogenic mutation nor loss of MMR proteins was detected. Therefore, our patient had not Lynch syndrome.

To my knowledge, this is the first report of HLA-B44 supertype-based immunotherapy in ovarian cancer. However, this study, like any case study, has notable limitations. It involves only one patient, and more research is needed to determine whether the principles found here apply to other patients.

## Conclusions

We report a PD-L1 negative, MSS, and TMB-L recurrent ovarian cancer patient with multiline chemotherapy who was highly sensitive to immunotherapy and had a long response. Our study suggests that the HLA-B44 supertype may be a potential biomarker for immunotherapy in ovarian cancer and immunotherapy is still attemptable in multidrug-resistant ovarian cancer. Although all traditional predictive factors of immunotherapy are negative, HLA-typing testing is also recommended. This hypothesis will need to be tested in larger randomized controlled trials, and there is a need to explore underlying cellular and molecular mechanisms.

## Data availability statement

The datasets presented in this article are not readily available because of ethical and privacy restrictions. Requests to access the datasets should be directed to the corresponding author/s.

## Ethics statement

The studies involving human participants were reviewed and approved by the ethics committee of Obstetrics and Gynecology Hospital of Fudan University. The patients/participants provided their written informed consent to participate in this study. Written informed consent was obtained from the individual(s) for the publication of any potentially identifiable images or data included in this article.

## Author contributions

LQY and MC conceived the study. MC and XL wrote the manuscript. XL evaluated and prepared images for the manuscript. TL from Precision Scientific (Beijing) Co, Ltd. performed WES. Each author participated in the patient’s medical care and revised the manuscript for the scientific content. All authors contributed to the article and approved the submitted version.

## Funding

This work was supported by grants from the National Natural Science Foundation of China (Grant No. 81802689).

## Acknowledgments

We gratefully thank the patient and her family for their kind cooperation and permission to publish this paper. We thank Jia Liu and Fenghua Ma (Obstetrics and Gynecology Hospital of Fudan University) for providing MRI images. **Figure 3** was created with BioRender.com.

## Conflict of interest

Author TL is employed by Precision Scientific (Beijing) Co, Ltd.

The remaining authors declare that the research was conducted in the absence of any commercial or financial relationships that could be construed as a potential conflict of interest.

## Publisher’s note

All claims expressed in this article are solely those of the authors and do not necessarily represent those of their affiliated organizations, or those of the publisher, the editors and the reviewers. Any product that may be evaluated in this article, or claim that may be made by its manufacturer, is not guaranteed or endorsed by the publisher.

## Abbreviations

OCCC, ovarian clear cell carcinoma; CR, completed response; PD-1, programmed cell death 1; OS, overall survival; RFS, recurrence-free survival; MSI, microsatellite instability; TMB, tumor mutational burden; EOC, epithelial ovarian cancer; ICIs, immune checkpoint inhibitors; ORR, objective response rate; NSCLC, non-small cell lung cancer; HLA-I, human leukocyte antigen class I; CA-125, carbohydrate antigen; PDS, primary debulking surgery; WES, whole exon sequencing; MSS, microsatellite stable; PD-L1, programmed death-ligand 1; MRI, magnetic resonance imaging; PD, progressive disease; TCR, T-cell receptor.
