# Supplementary material for: A durable response to programmed cell death 1 blockade in a multidrug-resistant recurrent ovarian cancer patient with HLA-B44 supertype: A case report
Source: Front Immunol. 2022 Oct 6;13:951422. doi: 10.3389/fimmu.2022.951422 (PMC9582938; doi:10.3389/fimmu.2022.951422)
Supplement: Supplementary file 1 [file DataSheet_1.pdf]

Supplementary Material

TABLE 1 Following radiologic results after immunotherapy (MRI)

Time	Location of lesions		
	In front of the inferior vena cava (cm)	Anterior to the tail of the pancreas (cm)	Around the head of the pancreas (cm)
2019-08*	4.5*2.0	2.0*1.5	1.9*1.1
2019-10	1.2*0.8	NA	1.0*0.8
2020-02	1.0*0.6	NA	1.0*0.9
2020-05	0.8*0.4	NA	0.8*0.5
2020-08	2.4*0.8	NA	0.8*0.4
2020-11	2.4*0.8	NA	0.9*0.6
2021-02	1.1*0.9	NA	0.9*0.7
2021-05	0.9*0.6	NA	1.0*0.9
2020-08	1.3*0.9	NA	1.0*0.4
2020-12	1.3*0.9	NA	1.1*0.5

*Baseline
